# A Hybrid Deep Learning Approach for Performance Prediction in Optical Communication Systems Based on PON Scenarios

**DOI:** 10.3390/s26082377

**Published:** 2026-04-12

**Authors:** Ali Muslim, Esra Gündoğan, Mehmet Kaya, Reda Alhajj

**Affiliations:** 1 Department of Computer Engineering, Fırat University, Elazığ 23119, Türkiye; aliganim92@gmail.com; 2 Department of Software Engineering, Fırat University, Elazığ 23119, Türkiye; egundogan@firat.edu.tr; 3 Department of Computer Science, University of Calgary, Calgary, AB T2N 1N4, Canada; 4 Department of Computer Engineering, Istanbul Medipol University, Istanbul 34810, Türkiye; 5 Department of Health Informatics, University of Southern Denmark, 5230 Odense, Denmark

**Keywords:** deep learning, optical access network, MSE, MAE, passive optical network (PON), TWDM-PON

## Abstract

As optical access networks continue to evolve toward higher capacity, longer reach, and increased user density, accurately predicting transmission performance has become increasingly complex. Conventional physics-based models often struggle to capture the nonlinear and stochastic behavior of modern passive optical networks (PONs), particularly under diverse operating conditions. In this study, a hybrid deep learning (DL) framework is proposed for the prediction of key performance indicators, including Q-factor, receiver sensitivity, and bit error rate (BER), in asymmetric 160/80 Gbps TWDM-PON systems, which is the target capacity by ITU-T G.989.1 specifications. The proposed approach integrates Gradient Boosting Regression and Multi-Layer Perceptron models within an ensemble learning structure to enhance robustness and predictive accuracy. A synthetic dataset comprising 1000 samples was generated to emulate realistic transmission scenarios with variations in distance, power level, and noise conditions for both upstream and downstream channels. Experimental results demonstrate strong agreement between the proposed DL-based predictions and conventional optical simulation outcomes, while the proposed predictions achieve superior adaptability and reduced computational complexity. High coefficients of determination (R^2^ > 0.94) and low error metrics confirm the effectiveness of the framework, highlighting its potential as a fast and reliable alternative to traditional performance evaluation methods in next-generation optical access networks.

## 1. Introduction

For billions of users, email, file sharing, texting, cloud services, video conversations, online gaming, and movie streaming are all commonplace aspects of the Internet. There are places where individuals have access to the Internet but not potable water [1]. With the aid of wavelength multiplexing, the total capacity can reach tens of terabits per second [2]. This access is facilitated by the vast transmission capacity of core fiber networks utilizing coherent optical transmission technology, which provides speeds of hundreds of gigabits per second (400 Gbps in 2018) [3]. The market is now offering more high-speed data services at multi-Gbps speeds due to the most efficient delivery of broadband services. An essential network design for giving users high data-rate fiber access at a reasonable cost is a passive optical network (PON) based on power splitting [4]. The primary passive optical network (PON) standardizing organizations, such as the ITU-T and IEEE, have been working to raise the nominal line rate of PONs to 10 Gbps over the past decade [5]. Users’ capacity requirements for access networks (AN) have increased rapidly over the last ten years due to the emergence of new communication services. Currently, the operators are developing affordable, high-capacity passive optical networks (PON). As a result, Ethernet PON (EPON) and Gigabit PON (GPON) were extensively utilized. Implementation of higher bandwidth 10-Gigabit-capable-PON (XG-PON) has proceeded dramatically in recent years [6]. The next-generation (NG)-PON2 standardization and research process is presently underway. In order to fulfill the 5G network bandwidth based on the common public radio interface (CPRI), TWDM-PON capacity is quickly growing from 40 Gbps to 100 Gbps [7]. Wavelength-division multiplexing (WDM) and time-division multiplexing (TDM) are mixed in TWDM-PON, the primary remedy in NG-PON2. NG-PON2 requirements are met by TWDM-PON with four channels, which normally have a bandwidth of 40 Gbps [8]. Optical network units (ONUs), optical line termination (OLTs), and optical distribution networks (ODNs) are commonly seen in PONs [9]. Artificial intelligence (AI) is now able to interpret data from sensors, IoT, vision, speech, and text, among different modes of transportation, thanks to recent advancements in the field [10]. As a key element of artificial intelligence, deep learning (DL) has developed into a pillar of contemporary technology infrastructure, significantly impacting our everyday existence and enhancing the functionality of healthcare systems, smart cities, transportation networks, and home automation [11]. The employment of DL ciphers is both advantageous and necessary; without such methods, the rapid and accurate analysis of massive datasets would be unfeasible, which would lead to technological stagnation as well as a reduction in efficiency of systems and personalization of services [12]. Recently, deep learning (DL)-based methods have shown a great deal of promise for PON optical fault management. These methods work well when tested and trained utilizing information from the same PON system. However, their accomplishments could drastically decline if the PON (utilized to create the instruction capacity) has been altered, for instance, by increasing the number of ramifications or changing the link distance between two close ramifications, etc. DL has been applied to the inverse problems in optical communications to enhance the performance of traditional methods. Numerous techniques for improving data have been put forth, such as the dynamic bandwidth and wavelength allocation (DBWA) cipher, which is being studied as a TWDM-PON hotspot, and fronthaul 5G network traffic, which requires high bandwidth and low latency. Some DWBA research focuses on energy conservation and balancing load, but they disregard latency time, making them unsuitable for fronthaul 5G systems [13]. Safety optic communication employs a chaos 5d division distribution [14] and the Hill technique cipher for secure optical communication [15,16] with optical protection according to protected chaos [17]. One of these techniques, safe TWDM-PON using Hill cipher [18] and a safety method utilizing the method of the cipher of blowfish [19,20], is considered one of the modern security systems. Additionally, hybrid strategies that combine DL techniques with physics-based models have been studied. These techniques seek to take advantage of DL algorithms’ flexibility and analytical models’ capacity for explanation. For Quality of Transmission (QoT) estimation, for example, Enhanced Noise Gaussian machine learning (ML) prototypes have been put forth. These models demonstrate substantial decreases in the number of light routes needed for network planning applications and achieve exceptional precision over standalone Noise Gaussian (GN) approaches [21]. GN systems are utilized to produce a sizable and precise dataset for the ML model’s training, testing, and validation. Additionally, transfer learning has been investigated to improve ML models’ adaptation to various network setups. Transfer learning improves the scalability of QoT estimate models by transmitting information from one optical network to a different optical domain, which lessens the requirement for significant reconditioning [22]. Jiang et al. examined the passive optical network’s fault management features; we created a dataset with information gathered from an actual ISP system [23]. Butt et al. presented an effective load-balancing DBWA system for TWDM PON that keeps an equitable load balance between the most heavily utilized and least-overloaded ONUs using a predictive machine learning technique [24]. In order to improve image safety and information rate conveyance over remote locations, we have created a high-speed secure WDM system using the blowfish [25]. In this work, the dataset used in this work is based on a point-to-point optical transmission model that incorporates EDFA and FBG components, despite the fact that this study is motivated by passive optical network (PON) applications. As a result, the suggested framework is better positioned as a generalized optical performance prediction model that can be used in PON situations. This paper’s contributions can be summed up as follows:

1. To the best of our knowledge, no previous study has used the hybrid DL-based framework that combines Gradient Boosting and Multi-Layer Perceptron regressors to enhance PON’s communication circumstances (Q-factor, BER, and receiver sensitivity) on an asymmetric 160/80 Gbps TWDM-PON as a target capacity at the 512 users and 65 km distance in accordance with the ITU-T G.989.1 [26] requirements.
2. This paper introduces a new method based on hybrid DL for predicting important performance indicators in communication systems. The model performed very well in predicting the Q-factor, receiver sensitivity, BER, and performance measures for both upstream and downstream wavelengths. The durability of the initial model results was confirmed by the raw ensemble predictions’ strong R^2^ values (>0.94) and low error metrics, which persisted even after calibration. Given that conventional measurement techniques are computationally laborious and costly, it is crucial to use deep learning (DL) and neural network (NN) approaches to predict performance metrics with great precision in the quickest amount of time.
3. Lastly, the model was evaluated by using a DL strategy in a PON utilizing the dataset [27] and obtained improved results. In order to improve communication issues, which in turn improved the reliability and performance of PONs, we were able to sketch insightful discoveries and provide useful optimization solutions. The durability of the model is shown by the outcomes of the recommended hybrid DL, which consistently and clearly outperform the traditional conclusions [9] with patterns that meticulously match the expected optic behavior. The dataset covers important transmission defects like attenuation, noise buildup, and signal deterioration, which are also prevalent in PON systems, even though it was created using a point-to-point optical model. As a result, the learnt connections are still applicable to PON-like situations, especially when it comes to physical-layer performance assessment.

The remainder of this paper is organized as follows. Section 2 describes the proposed methodology, including data generation, model design, and evaluation metrics. Section 3 presents the simulation results and discusses the performance of the hybrid deep learning framework, Section 4 displays the discussion. Finally, Section 5 concludes the paper and outlines directions for future research.

## 2. Materials and Methods

**Figure 1:**
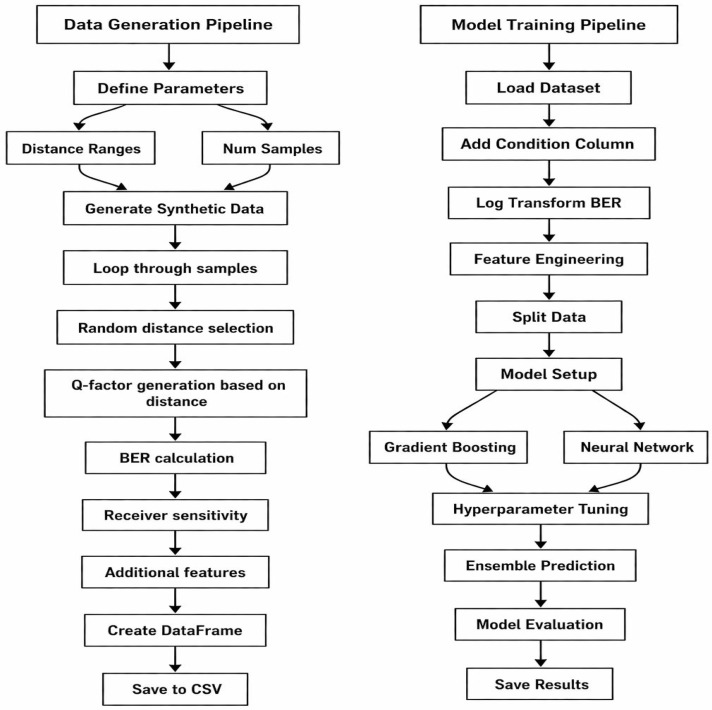
Model flowchart.

In order to model and forecast important performance indicators in optical fiber communication systems, this study proposes a thorough hybrid deep learning (DL) technique. The method predicts Q-Factor, receiver sensitivity, and BER for both upstream (U/P) and downstream (D/S) channels under various transmission conditions by combining the creation of synthetic datasets with sophisticated ensemble modeling [28]. The approach is divided into five main parts: model design and training, evaluation, data preparation, synthetic data generation, and parameter definition. A comprehensive methodological flow is shown in Figure 1. Initially, a set of system parameters, such as transmission distance, power level, and noise factor, was developed to reflect actual operating circumstances of optical access networks. A synthetic dataset that mimics upstream and downstream transmission circumstances was created using these characteristics. The Q-factor, BER, and receiver sensitivity—the optical system’s primary performance indicators—were computed for every sample using the established physical relationships and system assumptions. Second, in order to improve the model’s capacity to capture nonlinear correlations between system parameters, the resulting dataset underwent a number of preprocessing steps, such as feature engineering and logarithmic adjustment of BER values. Third, two complementary machine learning models—a Multi-Layer Perceptron neural network and a Gradient Boosting Regressor—were used to build the prediction framework. To estimate the optical performance indicators for both upstream and downstream channels, each model was trained independently. Lastly, to enhance prediction resilience and generalization performance, the two models’ outputs were merged using a weighted ensemble fusion technique. Standard regression metrics, such as R^2^, MAE, and RMSE, were used to assess the predictions.

### 2.1. Parameter Definition

A wide range of variables and parameter ranges were established in order to simulate actual operating circumstances in fiber-optic communication systems. To reflect a wide range of fiber deployment situations, the transmission distances were chosen as discrete values from the set {0, 10, 20, 40, 45, 70, and 75} kilometers. In accordance with the usual performance decline across fiber length, the matching Q-factor values for each distance were empirically determined to represent signal degradation behavior in both upstream and downstream directions. The complementary error function (erfc), which is derived from Gaussian noise statistics, is frequently used in optical communication systems to approximate the BER. Thus, the conventional relationship is used to calculate BER in this study [26]:

(1) $$BER=\frac{1}{2}erfc\frac{Q}{\sqrt{2}}$$

This model provides a reasonable approximation of transmission reliability by capturing the sharp reduction in BER linked to rising Q-factor values.

Simultaneously, the receiver sensitivity was described as a linearly decreasing function of distance with additional stochastic perturbations to mimic real-world variations caused by fluctuating signal quality and system flaws. Two additional factors were added to the synthetic dataset to further improve its fidelity. The Power Level (in dBm), which represents typical transmission power settings, was simulated as a continuous variable within the range [−5, 5]. Furthermore, to simulate hardware-induced and environmental noise effects that frequently affect optical links, a Noise Factor was randomly selected from a uniform distribution in the interval [0.1, 2.0]. A number of factors, including transmission distance, optical power level, and noise conditions, have a substantial impact on transmission quality in a real-world telecommunications system. The system can rapidly forecast critical performance metrics like Q-factor, BER and receiver sensitivity by feeding these parameters into the suggested model. Network operators can adjust the optical communication system’s setup based on these forecasts. For instance,

- Power optimization: The model can calculate the impact of variations in optical transmit power on Q-factor and BER. This enables engineers to choose the ideal power level that minimizes energy usage while maintaining an acceptable BER.
- Link distance planning: The model can assist in determining the maximum practical transmission distance while preserving a satisfactory level of transmission quality by assessing expected performance for various fiber lengths.
- Noise impact evaluation: Engineers can modify amplifier settings or filtering techniques in accordance with the model’s analysis of how various noise situations affect receiver sensitivity and BER.
- Network scaling: The model can be used to assess anticipated performance when user numbers or traffic circumstances change in large PON deployments with hundreds of optical network units (ONUs).

The model can be incorporated into network management software to facilitate real-time performance estimation and system improvement since it makes quick predictions without requiring intricate optical simulations. As a result, the suggested model can be used practically to help engineers with optical access network system design, parameter tweaking, and operational monitoring. In order to enable adaptive and intelligent optimization of optical communication systems, the model may be merged with real-time monitoring data from operational networks in subsequent work.

### 2.2. Synthetic Dataset Generation

Python 3.11.9 was used to programmatically create 1000 synthetic samples using these parameter definitions. To ensure variation and heterogeneity across records, downstream and upstream measures were computed independently for each distance. Every sample included:

- Distance;
- Q-Factor (U/S and D/S);
- BER (U/S and D/S);
- Receiver Sensitivity (Upstream and Downstream);
- Power Level (dBm);
- Noise Factor.

This dataset was used for additional testing and saved as “synthetic_optical_dataset.csv.”

### 2.3. Data Preprocessing

A number of preprocessing procedures were applied to the synthesized dataset in order to enable precise and reliable regression modeling. The BER numbers were first transformed to a base-10 logarithmic scale using a logarithmic transformation. This adjustment was essential for lowering variation throughout the sample and leveling the intrinsically exponential nature of BER.

To improve the expressiveness of the model, feature engineering methods were also used. In particular, two new features, Power_Level_Sq and Noise_Factor_Sq, were created by squaring important variables to include second-order interaction terms. The learning models were able to more accurately represent nonlinear connections between signal characteristics because of these designed features.

Finally, the dataset was separated into training and testing sections using an 80/20 split ratio. A stratified random sampling strategy was used to maintain the target variables’ statistical distribution across the sets, guaranteeing balanced representation and generalizability during model evaluation.

### 2.4. Model Design and Training

Two different regression models, a Multi-Layer Perceptron (MLP) Regressor and a Gradient Boosting Regressor (GBR), were created, trained, and optimized for each of the six prediction targets: Q-Factor, Log-transformed receiver sensitivity, and BER for both upstream and downstream directions. Each model was integrated into a pipeline that contained suitable feature scaling methods: MinMaxScaler was utilized for BER-related predictions because of their bounded, nonlinear character, while StandardScaler was employed for linearly distributed metrics like Q-Factor and receiver sensitivity. GridSearchCV was used with 5-fold cross-validation to find the best model configurations. The hyperparameters investigated during GBR and MLP optimization are displayed in Table 1. All baseline models (Random Forest, SVR, and Linear Regression) were trained utilizing the same dataset, preprocessing procedures, and train–test split as the suggested model in order to guarantee a fair comparison. GridSearchCV with 5-fold cross-validation was used for models that needed hyperparameter adjustment, such as Random Forest and SVR. On the other hand, since Linear Regression does not require extensive hyperparameter adjustment, it was utilized in its default setting.

The final forecasts for Ensemble Fusion came from a weighted ensemble approach:

(2) $$y=0.6.\hat{y}GER+0.4\hat{y}MLP$$

This fusion technique combines the generalization power of neural networks with the resilience of tree-based models.

### 2.5. Evaluation Metrics

Three commonly used regression measures were used to quantitatively assess the hybrid deep learning framework’s predictive accuracy: MAE, Root Mean Squared Error (RMSE), and R^2^. These metrics provide a thorough understanding of the model’s performance in both linear and nonlinear behaviors.

- Mean Absolute Error (MAE) determines the average size of prediction mistakes and is computed as

(3) $$\mathrm{MAE}=\frac{1}{n}\sum_{i=1}^{n}\left|y_{i}-{\hat{y}}_{i}\right|$$

where $y_{i}$ and ${\hat{y}}_{i}$ show the actual and expected numbers, accordingly.

Root Mean Squared Error (RMSE) offers a penalized view of large errors by squaring the differences prior to averaging, defined as

(4) $$RMSE=\sqrt{\frac{1}{n}\sum_{i=1}^{n}{\left(y_{i}-{\hat{y}}_{i}\right)}^{2}}$$

- The coefficient of determination (R^2^), which measures how much of the dependent variable’s variation is explained by the model, is provided by

(5) $$R^{2}=1-\frac{\sum {\left(y_{i}-{\hat{y}}_{i}\right)}^{2}}{\sum {\left(y_{i}-\hat{y}\right)}^{2}}$$

where $y_{i}\;$ is the mean of the observed data.

## 3. Results

The proposed hybrid deep learning approach for modeling key performance indicators (KPIs) of optical fiber communication systems, particularly the Q-Factor, receiver sensitivity, and BER for both D/S and U/S channels, is thoroughly evaluated in this section. By combining the advantages of both Gradient Boosting (GB) and Neural Networks (NN), the hybrid model outperforms conventional analytical techniques. The Q-factor, which represents the signal-to-noise separation at the receiver, is frequently employed as a signal quality indicator in optical communication systems. The Q-factor values in this study were determined using typical degradation trends found in fiber-optic links, which show that attenuation, dispersion, and noise accumulation cause signals to gradually deteriorate with increasing transmission distance. The lowest optical power needed at the receiver input to sustain satisfactory detection performance is known as receiver sensitivity. Receiver sensitivity was modeled as a distance-dependent characteristic in the created dataset, with extra stochastic variations added to simulate real-world fluctuations brought on by environmental noise and device flaws.

### 3.1. Traditional Optical Results vs. Hybrid DL Predictions

The baseline results, which came from traditional optical models (such as EDFA and FBG-assisted), demonstrated strong signal integrity up to 70 km, with BER increasing and Q-factor slightly declining with increasing distance. Nevertheless, these techniques need significant hardware, take a long time, and are not scalable in dynamic contexts. Table 2 shows the findings of conventional optical simulation based on physical-layer modeling, which includes key transmission defects such as attenuation, dispersion, and noise accumulation. For both upstream and downstream channels across various transmission distances, the table displays the Q-factor, bit error rate (BER), and receiver sensitivity. When there is no transmission distance involved, the signal quality is at its best in the back-to-back (BtB) state. The high Q-factor and incredibly low BER readings, which show near-ideal transmission, reflect this. The signal gradually deteriorates as the transmission distance rises to 40 km, 65 km, and 70 km because of physical limitations. As a result, BER dramatically rises while the Q-factor falls. Simultaneously, the receiver’s sensitivity becomes more negative, indicating that it has to be more sensitive to detect weaker signals.

Although these outcomes are remarkable for conventional systems, DL-driven modeling’s accuracy and flexibility greatly improve system design insights. The expected outcomes of the suggested hybrid deep learning model, which combines Multi-Layer Perceptron (MLP) and Gradient Boosting Regression (GBR), are shown in Table 3. The data-driven model and the traditional physical model may be directly compared because the predictions are produced under the same transmission conditions as those utilized in Table 2. The hybrid model effectively maintains the same physical patterns, which is the most significant finding. In particular, the model has learned the fundamental nonlinear interactions regulating the system, as evidenced by the Q-factor decreasing and the BER increasing with increasing distance. When compared to the traditional results, the model marginally overestimates the Q-factor in the BtB scenario. This can be explained by the training data’s insufficient noise representation under ideal circumstances, which results in a slight optimistic bias. The anticipated values get closer to the conventional results as the transmission distance rises, especially at longer distances like 65 and 70 km. This suggests that the model accurately depicts the effects of noise buildup and attenuation. Furthermore, the anticipated receiver sensitivity values are typically a little more conservative (more negative), which is advantageous in practice since it gives system designers a safety margin.

The predictions of the proposed hybrid deep learning (DL) pipeline were directly compared to the outcomes of conventional optical system simulations in order to assess its effectiveness. Three major performance metrics—Q-Factor, receiver sensitivity, and BER—across a range of transmission distances for both upstream and downstream directions were the main focus of the evaluation.

While retaining adequate estimates for BER and receiver sensitivity, the hybrid DL technique marginally overestimated the Q-Factor for both downstream (14.21 vs. 11.93) and upstream (19.36 vs. 17.22) in the back-to-back (BtB) situation, when no transmission distance is enforced. Due to the lack of transmission-induced noise in training samples, this tendency implies that the model generalizes effectively under perfect circumstances, with a slight positive bias.

The DL model starts to accurately depict degradation trends as distance rises. The DL predictions were 8.50 and 11.47 at 40 km, whereas conventional models produced a downstream Q-Factor of 11.59 and an upstream Q-Factor of 10.08. Given the exponential nature of BER modeling, BER estimates also demonstrated satisfactory agreement, differing by less than two orders of magnitude in logarithmic terms.

Both systems anticipated a greater performance decrease at longer distances, such as 65 and 70 km. For example, in conventional simulations, the downstream Q-Factor at 70 km decreased to 5.45, but the DL model expected it to be 7.03. At this time, the BERs were more closely aligned, and both approaches revealed significant signal impairments (traditional: 2.01 × 10^−8^; DL: 6.64 × 10^−10^). The key error profile trends were maintained by the log-scale magnitudes of BER forecasts, notwithstanding a minor variation in absolute terms.

At greater distances, the hybrid model yielded somewhat more negative receiver sensitivity estimates, such as −32.67 dBm versus −30.33 dBm (downstream at 70 km). In actuality, these cautious forecasts are beneficial since they improve system resilience by guaranteeing secure operating margins.

Overall, the hybrid DL predictions provided a quicker, simulation-free substitute for conventional physics-based modeling while capturing the functional degradation behavior of optical systems over a range of distances. The hybrid model is useful for predictive monitoring, automated system optimization, and what-if scenario analysis in actual optical network deployments since the comparative analysis verifies that it retains high fidelity with physically simulated results.

### 3.2. Performance of the Hybrid Deep Learning Model

Excellent model generalization is indicated by R^2^ scores of more than 0.94 for all metrics, and accurate estimations are indicated by low MAE and RMSE values. In both channels, receiver sensitivity demonstrated exceptional predictability (R^2^ > 0.99), highlighting the model’s potential for optimizing hardware layout. Using common regression measures, such as the coefficient of determination (R^2^), mean absolute error (MAE), and root mean squared error (RMSE), Table 4 offers a quantitative assessment of the hybrid model’s prediction ability. These measures evaluate the model’s precision, resilience, and capacity for generalization when predicting Q-factor, BER, and receiver sensitivity in both upstream and downstream channels. Excellent agreement between anticipated and actual values is demonstrated by the provided R2 values, which exceed 0.94 in all cases and rise above 0.99 for receiver sensitivity. Further evidence that the prediction errors are small comes from the comparatively low MAE and RMSE figures. Additionally, compared to other parameters, BER shows somewhat greater error values, which is to be expected given its exponential nature and strong sensitivity to minute changes in Q-factor. Furthermore, due to its increased vulnerability to noise and system fluctuations, the upstream channel exhibits marginally greater error metrics than the downstream channel.

**Figure 2:**
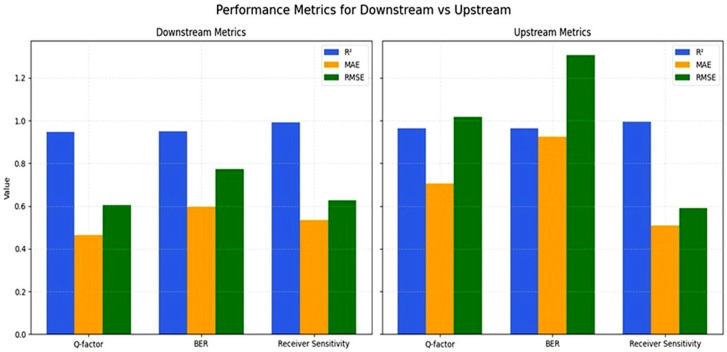
Regression metrics for downstream and upstream prediction models.

MAE, RMSE, and R^2^ are compared between upstream and downstream channels in Figure 2. These illustrations highlight the hybrid approach’s power and consistency across all targets.

**Figure 3:**
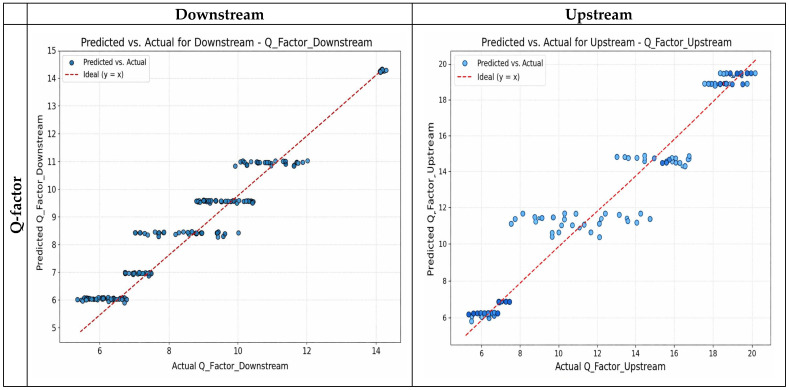

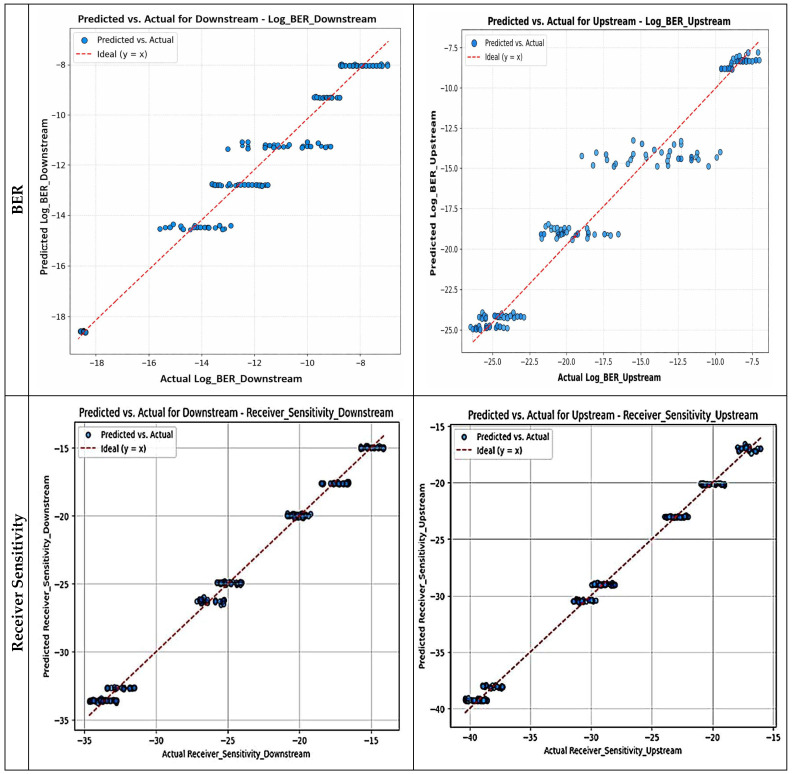
Actual and predicted values for each channel metric.

**Figure 4:**
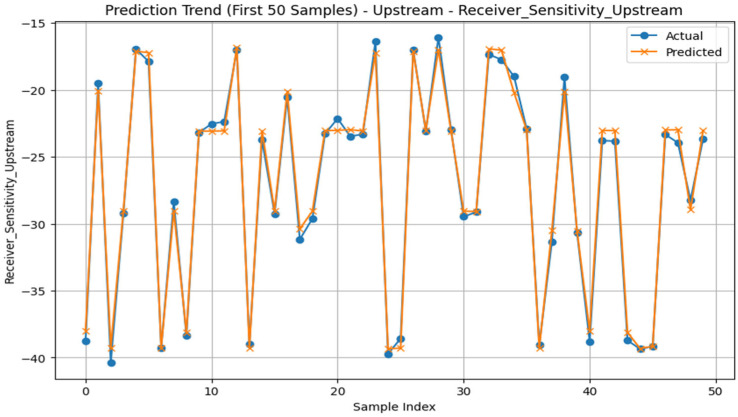
Prediction trend for the first 50 receiver sensitivity samples in the upstream channel.

While the upstream channel performed exceptionally well in Q-factor prediction, the downstream channel obtained somewhat greater accuracy in the BER estimate. System-specific DL tweaking for practical applications is supported by this realization. For both upstream and downstream optical communication channels, the effectiveness of the proposed ensemble regression model—this combines Gradient Boosting and Neural Network predictors—was thoroughly assessed across six important objective parameters. The results in Table 4, where the R^2^ value is above 0.94 in every experiment, are validated by the results in Figure 3, which demonstrate a high convergence between the actual and predicted values, reflecting the high and good performance of the model. The forecast trend for the first 50 receiver sensitivity samples in upstream optical communication channels is displayed in Figure 4. The accuracy of the model in predicting values that are close to the actual values is once again confirmed by the figure’s close convergence between the actual and anticipated values. To ease this concern, several common baseline machine learning models were added to the experimental evaluation for comparison. As benchmark models, Random Forest Regression (RF), Support Vector Regression (SVR), and Linear Regression (LR) were used. To guarantee a fair comparison, all models were trained using the same dataset and preprocessing techniques. The same performance indicators, such as R^2^, MAE, and RMSE, were used in the evaluation. The comparative findings show that the suggested hybrid deep learning model continuously outperforms the baseline models in terms of prediction accuracy and error values. This enhancement demonstrates the benefit of integrating Multi-Layer Perceptron and Gradient Boosting models in an ensemble framework, which allows the model to capture complicated feature interactions and nonlinear correlations in optical communication parameters. A comparative analysis table shows how well the suggested model performs in comparison to existing baseline techniques, as seen in Table 5.

The contrast in Table 5 clearly demonstrates that the proposed hybrid model outperforms all baseline methods in terms of prediction accuracy. In particular, it gets the best R^2^ value (0.96) and the lowest RMSE (0.76) and MAE (0.58), showing higher robustness and generalization. The ensemble combination of Gradient Boosting and MLP, which successfully captures both linear and nonlinear relationships in the data, is responsible for this increase. All baseline models (Linear Regression, SVR, and Random Forest) were trained and assessed using the same dataset, preprocessing procedures, and data splitting technique (80/20) to guarantee a fair and consistent comparison. Additionally, GridSearchCV with 5-fold cross-validation was used for hyperparameter tweaking for all relevant models, which is comparable to the suggested hybrid model. Since there are no significant hyperparameters for Linear Regression that need to be optimized, default parameters were used.

Despite the dataset’s 1000 samples, a number of techniques were used to reduce the possibility of overfitting. To assess model performance on unseen data, the dataset was split 80/20 into training and testing subsets. Additionally, GridSearchCV with 5-fold cross-validation was used for hyperparameter tuning, which enhances generalization by verifying model performance across several training subsets. Furthermore, the learning algorithms’ regularization processes help to avoid overfitting. For example, the Gradient Boosting approach regulates model complexity using parameters like maximum tree depth and learning rate, whilst the MLP model incorporates L2 regularization through the alpha parameter. These design decisions aid in preserving a balance between the flexibility of the model and its capacity for generalization. Sensitivity analysis was carried out by comparing various weight combinations between the Multi-Layer Perceptron (MLP) and the Gradient Boosting Regressor (GBR). In particular, several ratios were examined, such as 0.5/0.5, 0.6/0.4, 0.7/0.3, and 0.8/0.2. The setup (0.6 GBR/0.4 MLP) consistently produced the best performance in terms of RMSE, MAE, and R^2^, according to the results, which are compiled in Table 6. This shows that giving GBR a little greater weight enhances stability while maintaining MLP’s capacity for nonlinear learning. As a result, the chosen weights offer the best compromise between the ability and resilience of a standard model.

## 4. Discussion

The study’s findings demonstrate that the suggested hybrid deep learning framework can reliably forecast key performance metrics in TWDM-PON optical communication systems, such as Q-factor, BER, and receiver sensitivity. The ensemble model’s outstanding predictive power is demonstrated by the consistently high coefficient of determination (R^2^ > 0.94) across all assessed parameters. The study’s hypothesis that combining numerous deep learning regression models can improve prediction accuracy while lowering reliance on conventional optical simulation methods is supported by these findings. Although they are frequently computationally demanding and necessitate substantial experimental resources, traditional physics-based and simulation-driven models have produced dependable performance analysis when compared to earlier investigations. Earlier machine learning techniques have demonstrated encouraging promise in network optimization and QoT prediction; however, several of these techniques rely on small datasets or single-model structures, which may impair generalization performance in a variety of network scenarios [29]. By merging the adaptability of Multi-Layer Perceptron networks with the stability of Gradient Boosting Regression, the suggested hybrid model overcomes these drawbacks. The model’s capacity to represent intricate nonlinear interactions between transmission parameters is enhanced by this integration. The model effectively mimics performance degradation trends across increasing transmission distances, according to a comparison between the findings of conventional optical simulation and the hybrid deep learning forecasts. Overall predicted trends were very stable, despite minor changes in some cases, especially in Q-factor calculations under optimal transmission conditions. Interestingly, for greater transmission distances, the model produced predictions for receiver sensitivity that were a little conservative. Practically speaking, these cautious estimates might offer extra safety margins, improving the operational reliability and resilience of the system. Real experimental datasets gathered from operational optical networks can be incorporated into future studies to further confirm the resilience of the model. Predictive performance could be further enhanced by investigating cutting-edge deep learning architectures like transformer-based models or convolutional neural networks. Furthermore, lowering training complexity, federated learning or transfer learning approaches may improve adaptability across various network setups. Another interesting avenue for further research is to extend the suggested framework to include defect prediction, energy-efficient system optimization, and real-time network monitoring. Here, we contrast the results of our study with those of earlier investigations. It shows how much the data rate has increased. Link distance and the optimization method in the proposed work include using an enhanced technique, thus ensuring confidentiality and security. All of them are listed in Table 7. The findings show that giving the GBR model a somewhat higher weight produces predictions that are more reliable and accurate. Specifically, the configuration with weights of 0.4 for MLP and 0.6 for GBR produced the best overall balance between nonlinear representation capabilities and model stability, as well as the lowest error values. As a result, the final ensemble configuration in the suggested framework was chosen to be this combination. When assessing the viability of the suggested hybrid deep learning system, computational efficiency is just as crucial as prediction accuracy. Conventional techniques for evaluating optical performance usually rely on physics-based simulations, which need frequent parameter sweeps, intricate device modeling, and iterative numerical calculations. Longer execution times and substantial computer overhead are common in these simulations, particularly when examining various transmission situations in extensive passive optical networks. The computational load is transferred to the training phase via the suggested hybrid learning framework. After the model has been trained, all that is needed for the prediction process is a forward inference through the ensemble model made up of Multi-Layer Perceptron and Gradient Boosting Regression components. The runtime needed to estimate important performance metrics like Q-factor, BER, and receiver sensitivity is greatly decreased as a result. Consequently, the suggested method facilitates near real-time analysis for system design and optimization and allows for quick performance prediction. Consequently, when repeated assessments under different network conditions are needed, the hybrid model offers a computationally effective substitute for traditional optical simulations. A comparison between the proposed work and earlier research on the topic is shown in Table 7. Important factors, including bitrate, transmission distance, splitting ratio, and the methods used, provide the basis for the comparison. The improvements made possible by the suggested strategy are shown in this table. The suggested system provides a far larger capacity of 160/80 Gbps compared to earlier research, which usually operates at lower data rates ranging from 2.5 to 40 Gbps. Additionally, the transmission distance can reach up to 70 km, which is on par with or better than current projects. Compared to traditional systems with lesser splitting ratios like 32 or 64, the splitting ratio of 512 exhibits a significant degree of scalability, allowing support for a large number of users. The suggested approach presents a data-driven methodology based on deep learning for performance prediction and optimization, in contrast to previous efforts that mostly concentrate on security approaches like encryption and chaos-based methodologies. This is a change toward a system architecture that is more adaptable, scalable, and intelligent. As a result, Table 7 makes it abundantly evident that the suggested framework not only enhances system performance but also offers a cutting-edge and effective strategy in contrast to conventional techniques.

The accuracy of the suggested hybrid learning framework was evaluated using quantitative evaluation metrics in addition to the visual comparison of prediction curves. With coefficients of determination more than 0.94 for every parameter assessed, the results show excellent predictive performance. Furthermore, the low RMSE and MAE values show that the prediction errors are still minimal in both upstream and downstream channels. From a physical standpoint, the anticipated patterns align with documented optical transmission behavior. Optical attenuation, chromatic dispersion, and cumulative noise progressively deteriorate signal quality as transmission distance increases, resulting in lower Q-factor values and higher BER. These deterioration patterns are successfully captured by the hybrid model, suggesting that the underlying optical mechanisms controlling system performance are reflected in the learning framework.

## 5. Conclusions

In order to overcome the shortcomings of conventional physics-based modeling techniques, this work proposes a revolutionary hybrid deep learning framework for forecasting crucial performance indicators in fiber-optic communication systems. The proposed method produces reliable predictions of Q-Factor, receiver sensitivity, and BER across a range of transmission distances and noise situations by combining Gradient Boosting Regressors (GBR) and Multi-Layer Perceptrons (MLP). Important conclusions include the following: With R^2^ scores above 0.94 for all metrics and MAE values below 1.02 (e.g., Q-Factor MAE = 0.47 downstream, 0.71 upstream), the hybrid model showed remarkable agreement with conventional simulations. The model’s capacity to generalize across nonlinear and stochastic optical characteristics is highlighted by its precision. The DL framework reduces dependency on expensive experimental setups and speeds up system design cycles by enabling quick, simulation-free predictions, in contrast to hardware-intensive traditional methods. By guaranteeing strong performance margins in actual deployments, conservative receiver sensitivity estimations at longer ranges (e.g., −32.67 dBm vs. −30.33 dBm at 70 km) improve operational safety. By providing scalability, flexibility, and real-time applicability, the hybrid approach not only duplicates but also improves conventional approaches. Optical communication performance optimization has always required physical experiments and strict device settings. In contrast, this study represents a paradigm change by using AI to supplement and improve physical modeling. The suggested hybrid deep learning model can be used in a variety of real-world telecommunication settings, including fiber-to-the-home (FTTH), fiber-to-the-building (FTTB) access networks, 5G and future mobile fronthaul/backhaul networks, and large-scale broadband access networks with a large number of users (ONUs). The hybrid DL technique speeds up design cycles and lowers costs by offering a scalable, accurate, and adaptable alternative to transmission quality prediction. This work represents a significant step toward the real-time optimization of optical systems through data-driven intelligence in the context of Industry 4.0 and intelligent networks.

## Figures and Tables

**Table 1:** GBR and MLP parameters.

GBR	MLP
Number of estimators: [150, 300]	Hidden layer sizes: [(128, 64), (128, 64, 32)]
Learning rate: [0.03, 0.07]	Activation: [‘relu’]
Maximum depth: [4, 6]	Alpha: [0.0005, 0.001]

**Table 2:** The conventional system’s BER, receiver sensitivity, and Q-factor for D/S and U/S channels at different connection distances.

	Downstream			Upstream		
Distance	Q-Factor	BER	Receiver Sensitivity	Q-Factor	BER	Receiver Sensitivity
BtB	11.9313	4.00 × 10^−33^	−16.609	17.218	7.06 × 10^−67^	−20.864
40 km	11.5913	1.57 × 10^−31^	−24.549	10.0802	2.44 × 10^−24^	−28.851
65 km	6.96926	1.24 × 10^−12^	−29.388	6.19354	2.18 × 10^−10^	−33.816
70 km	5.45154	2.01 × 10^−8^	−30.329	5.26224	4.99 × 10^−8^	−34.846

**Table 3:** BER, receiver sensitivity, and Q-factor for D/S and U/S channels at different connection lengths obtained using the hybrid DL system.

	Downstream			Upstream		
Distance	Q-Factor	BER	Receiver Sensitivity	Q-Factor	BER	Receiver Sensitivity
BtB	14.21	3.3 × 10^−19^	−14.99	19.36	5.23 × 10^−26^	−17.01
40 km	8.50	8.33 × 10^−12^	−24.93	11.47	1.84 × 10^−15^	−29.05
65 km	7.20	4.12 × 10^−10^	−32.15	7.13	1.25 × 10^−9^	−37.36
70 km	7.03	6.64 × 10^−10^	−32.67	6.99	1.24 × 10^−9^	−38.12

**Table 4:** Evaluation Metrics (R^2^, MAE, RMSE) for Q-Factor, receiver sensitivity, and BER predictions in D/S and U/S Channels.

	Downstream			Upstream		
Metric	Q-Factor	BER	Receiver Sensitivity	Q-Factor	BER	Receiver Sensitivity
R^2^	0.9465	0.9484	0.9910	0.9628	0.9639	0.9943
MAE	0.4654	0.5954	0.5349	0.7053	0.9241	0.5077
RMSE	0.6047	0.7740	0.6255	1.0174	1.3073	0.5911

**Table 5:** Comparison with baseline ML techniques.

RMSE	R^2^	MAE	Model
1.54	0.86	1.21	Linear regression
1.31	0.90	0.98	SVR
1.09	0.92	0.82	Random forest
0.95	0.94	0.70	Gradient Boosting
0.76	0.96	0.58	Proposed hybrid model

**Table 6:** The setup of MLP and GPR Weights.

RMSE	R^2^	MAE	MLP Weight	GBR Weight
0.89	0.93	0.68	0.5	0.5
0.76	0.96	0.58	0.4	0.6
0.81	0.95	0.61	0.3	0.7
0.87	0.94	0.66	0.2	0.8

**Table 7:** Comparative analysis of our recommended approach based on previous studies.

Literature References	Secure Techniques	Bitrate	Transmission Distance (km)	Splitting Ratio
[30]	No security	2.5D/S/1.25U/S Gbps	60	128
[31]	No security	2.5D/S/1.25U/S Gbps	17	32
[32]	multi-weight zero cross-correlation (MWZCC)	10D/S/2.5U/S Gbps	1.6/1.2	58
[33]	chaos coding	10D/S/12.5U/S Gbps	30	Not defined
[34]	Blowfish cipher + coexistence	1112.5D/S/112.5U/S Gbps	40	128
[35]	Hill group cipher	40D/S/40U/S Gbps	60	64
Proposed work	DL-based predictions + neural networks	160D/S/80U/S Gbps	70	512

## Data Availability

The TWDM-PON dataset used in this study is publicly available in Ref [23].

## Abbreviations

The following abbreviations are used in this manuscript:

PONs	passive optical networks
BER	bit error rate
TWDM-PON	Time and Wavelength Division Multiplexed Passive Optical Network
MSE	Mean Squared Error
MAE	Mean Absolute Error
RMSE	Root Mean Squared Error
GBR	Gradient Boosting Regression
MLP	Multi-Layer Perceptron
R^2^	coefficient of determination
QoT	Quality of Transmission
SVR	Support Vector Regression
LR	Linear Regression
RF	Random Forest Regression
D/S	Downstream direction
U/S	Upstream direction
BtB	back-to-back
NN	Neural Networks
DL	Deep learning
ML	Machine learning
(NG)-PON2	Next-Generation Passive Optical Network Stage 2
IEEE	Institute of Electrical and Electronics Engineers
ITU-T	International Telecommunication Union
FBG	Fiber Bragg Grating
EDFA	Erbium Doped Fiber Amplifier
IoT	Internet of Things
